# Linggui Zhugan Formula Improves Glucose and Lipid Levels and Alters Gut Microbiota in High-Fat Diet-Induced Diabetic Mice

**DOI:** 10.3389/fphys.2019.00918

**Published:** 2019-07-23

**Authors:** Rui Wu, Dandan Zhao, Ran An, Zhufeng Wang, Yuxiu Li, Bai Shi, Qing Ni

**Affiliations:** ^1^ Department of Endocrinology, South Area of Guang’anmen Hospital, China Academy of Chinese Medical Sciences, Beijing, China; ^2^ Chinese Medicine School, Beijing University of Chinese Medicine, Beijing, China

**Keywords:** Chinese medicine, obesity, glucose, lipid, gut microbiota

## Abstract

**Background:** The gut microbiota plays important roles in the occurrence and development of obesity and diabetes through participating in nutrient absorption and metabolism. Microecological regulation is likely to be key to understanding the effects of Chinese medicine. The Linggui Zhugan (LGZG) formula is a well-known Chinese medicine for controlling obesity in the clinic. However, its pharmacological effects and mechanism of action in diabetes require further exploration.

**Objective:** To evaluate the effects of LGZG on body weight, glycemic control, lipid levels, and gut microbiota in high-fat diet-induced diabetic mice.

**Methods:** High-fat diet-induced diabetic mice were subjected to an 8-week protocol of LGZG administration. We then evaluated the pharmacological effects of LGZG and its influence on gut microbes in fecal samples using the 16S rRNA-based microbiome profiling technique.

**Results:** LGZG administration significantly reduced body weight and body fat mass in diabetic mice. Compared with the high-fat diet control group, LGZG favorably influenced blood glucose control, decreased blood glucose levels, and increased glucose tolerance, accompanied by an improvement in lipid metabolism. Furthermore, the global community composition and relative abundance of many taxa differed between mice fed chow or a high-fat diet. As expected, LGZG supplementation altered the general community structure of gut microbiota, the Firmicutes/Bacteroidetes ratio, and the relative abundance of certain bacteria, such as *Bacteroides, Lactobacillus, Oscillospira,* and *Helicobacter*.

**Conclusion:** LGZG effectively controlled obesity and relieved insulin resistance, which may be closely related to its impact on gut microbiota.

## Introduction

Metabolic disorders, such as obesity and type 2 diabetes mellitus (T2DM), have attracted global attention because of their increasingly high prevalence. One key pathogenic process linking obesity to T2DM is insulin resistance, which occurs throughout the development of T2DM. The development of obesity and T2DM is related to many factors, including genetic and environmental factors. The gut, a very important environmental factor that influences T2DM development (Sircana et al., 2018), has become a research subject of great interest in recent decades.

It is generally accepted that a strong relationship exists between the gut microbiota composition and specific metabolic disorders, such as obesity and T2DM (Canfora et al., 2019). Germ-free mice were shown to become obese upon the transfer of microbiota from obese mice, and vice versa (Turnbaugh et al., 2006). Additionally, the transfer of microbiota from obesity-prone, but not obesity-resistant, rats to germ-free mice replicates the obesity-prone phenotype (Duca et al., 2014). Obesity is a major contributor to T2DM. Similarly, the relationship between T2DM and the gut is also very close. Studies in both diabetic patients and mice have confirmed that differences in gut microbiota partly explain the incidence and progression of diabetes (Qin et al., 2012; Greiner et al., 2014). A previous study showed a significant increase in not only the diversity of the intestinal flora but also the glucose uptake and insulin sensitivity of obese individuals 6 weeks after fecal bacterial transplantation from lean subjects (Vrieze et al., 2012). Furthermore, the transfer of gut microbiota from diabetes-protected MyD88-deficient nonobese diabetic mice reduced insulitis and significantly delayed the onset of diabetes (Peng et al., 2014).

Regarding the mechanisms underlying the link between the gut microbiota and its host, data demonstrate that the gut microbiota communicates with host organs through several pathways, such as nervous routes (e.g., the enteric nervous system, vagus nerve, and brain) and endocrine routes [e.g., glucagon-like peptide-1 (GLP-1), peptide YY (PYY), and endocannabinoids]. Moreover, metabolites from gut microbiota [e.g., short-chain fatty acids (SCFAs), serotonin, bile acids, and bioactive lipids] regulate several physiological and pathological processes in the host, including effects on appetite regulation, energy intake, energy expenditure, and lipid oxidation (Rastelli et al., 2018; Canfora et al., 2019). In addition, researchers also found that intestinal gut disorders promote the production of cytokines, metabolic endotoxins, inflammatory factors, etc., in the body (Marchesi et al., 2016). Although the mechanisms still need further exploration, the gut is generally accepted to influence insulin sensitivity and glucose and lipid metabolism. Therefore, regulation of the gut could counter the development of T2DM.

Linggui Zhugan (LGZG) formula, a classic traditional Chinese medicine prescription, has been widely used to prevent and treat various diseases with the syndrome of Qi deficiency and dampness accumulation. LGZG contains four herbs: Fuling (Poria), Guizhi (Ramulus Cinnamomi), Baizhu (Rhizoma Atractylodis Macrocephalae), and Gancao (Radix Glycyrrhizae). Despite increasing evidence from both clinical and epidemiological studies in humans (Chen et al., 2011; Yang et al., 2014; Liang and Wang, 2016) showing that LGZG is effective in reducing the body weight of individuals with obesity or metabolic syndrome, the effect of LGZG on insulin resistance and its underlying mechanism remain largely unclear. Modern pharmacological studies have indicated that the effect of LGZG on hyperlipidemia may be associated with its influence on hemodynamic status (Zhang et al., 2003). However, little attention has been paid to the potential impact of LGZG on diabetic mice. The aim of this study was to evaluate the effects of LGZG on high-fat (HF) diet-induced diabetic mice and to further explore whether imbalances in intestinal microflora can be improved by LGZG.

## Materials and Methods

### Animals and Diet

Six-week-old male C57BL/6 J Spf mice were obtained from SPF Biotechnology Co., Ltd. (Beijing) [Certificate No. SCXK (Jing) 2016-0002] and housed individually in ventilated cages under clean level conditions [Certificate No. SCXK (Jing) 2016-0038] in a controlled environment [inverted 12-h daylight cycle with constant temperature (23 ± 2°C) and humidity (55 ± 10%)] with free access to food and water. The mice were fed a standard chow diet (10% kcal from fat, set as the normal control group, *n* = 10) or a HF diet (60% kcal from fat, *n* = 55) for 12 weeks. All diets, whose compositions are listed in Supplementary Material S1, were manufactured by Mediscience Co., Ltd. (Jingsu, China).

### Drugs

LGZG contains four drugs: Fuling (Poria), Guizhi (Ramulus Cinnamomi), Baizhu (Rhizoma Atractylodis Macrocephalae), and Gancao (Radix Glycyrrhizae). The extracts of the above drugs were purchased from Jiangyin Tianjiang Pharmaceutical Co., Ltd. (Jiangyin, China). Then, the extracts were mixed and dissolved in water at the ratio of 4:3:3:2 for oral administration to mice. A sample of the extract mixture was diluted with methanol, filtered through a 0.45-μm filter, and subjected to high-performance liquid chromatography (HPLC) analysis to characterize the chemical composition for quality control of the LGZG mixture. The main signals in the chromatogram were identified, and the chromatogram is shown in Supplementary Material S2.

### Experimental Design

Forty diabetic mice in the HF group with a fasting blood glucose (FBG) over 7.0 mmol/L were selected and randomly subdivided into the following four groups with 10 mice per group: LGZGH (3.6 g/kg BW), LGZGL (1.8 g/kg BW), metformin (75 mg/kg/d), or vehicle (equal volume of saline, set as the HF control group). The treatments were administered by gavage to mice in each respective group daily for 8 weeks. The above dosages of the drugs were compatible with human use. During the experimental period, body weight and FBG levels were assessed weekly. Oral glucose tolerance tests (OGTTs) and insulin tolerance tests (ITTs) were performed at week 8. Body fat mass was measured at the end of the experiment. Feces were collected at 8 weeks to analyze microbiota. Blood samples were collected, and the serum was used for further biochemical analyses, including of glycosylated hemoglobin (HbA1C), insulin, glucagon, lipids [total cholesterol (TC), triglyceride (TG), high-density lipoprotein cholesterol (HDL-C), and low-density lipoprotein cholesterol (LDL-C)] and free fatty acids (FFAs). The homeostasis model of insulin resistance (HOMA-IR) was calculated using the formula HOMA-IR = fasting serum insulin (mU/ml) × fasting plasma glucose (mM)/22.5. The animal experiments were performed in accordance with the conventional guidelines for the Care and Use of Laboratory Animals from the Committee for Animal Experiments of the National Center. The protocol was approved by the Animal Ethics Committee of Beijing University of Chinese Medicine.

### Body Fat Mass

The body fat mass of the mice was assessed by nuclear magnetic resonance (NMR) scanning imaging (MRI; EchoMRI-100 for mice, Echo Medical System, Houston, USA) at the end of the experiment (week 8). Total fat mass was measured, and the fat content was calculated (fat mass weight/body weight × 100%).

### Oral Glucose Tolerance Test and Insulin Tolerance Test

At 8 weeks, animals were fasted overnight, and an oral glucose load was administered at 2 g/kg body weight. Blood glucose levels were measured from the tail vein before and at 15, 30, 60, 90, and 120 min after glucose administration. Glucose tolerance was evaluated by calculating the areas under the curve (AUCs).

The insulin tolerance test (ITT) was performed by injecting 1 U/kg human insulin (Insulatard; Novo Nordisk, Bagsvaerd, Denmark) after a 6-h fast 3 days before the end of the experiment. Blood glucose levels were measured at 0, 30, 60, 90, and 120 min after insulin injection. Glucose tolerance was evaluated by calculating the AUCs.

### Biochemical Assay

Profiles of blood lipids, including serum TC, TG, LDL-C, and HDL-C, were obtained using biochemistry reagent kits (Nanjing Jiancheng Biology Engineering Institute, Nanjing, China) and an automated biochemical analyzer (Hitachi, Tokyo, Japan). Blood plasma FFA content was measured by an enzymatic assay (Clinimate NEFA, Tokyo, Japan). Plasma insulin and glucagon levels were assessed using an insulin ELISA kit (Shibayagi, Gunma, Japan) and glucagon ELISA kit (Shibayagi, Gunma, Japan), respectively, according to the manufacturer’s instructions.

### DNA Extraction From Mouse Fecal Samples

DNA was extracted from feces using a TIANamp Stool DNA Kit (TIANGEN Biotech, Beijing, China). The DNA used for library preparation was quantitated and subjected to quality control with a NanoVue Biophotometer Plus (Bio-Rad, Hercules, CA), and DNA extracts were stored at −20°C until use.

### Polymerase Chain Reaction Amplification

Primers 341f (5′-CCTAYGGGRBGCASCAG-3′) and 806r (5′-GGACTACNNGGGTATCTAAT-3′) were used to amplify the V3-V4 region of bacterial 16S rRNA. Illumina sequencing libraries (Zhang et al., 2019) were prepared using the one-step PCR in a 25-μl reaction mixture containing 25 ng of input DNA, 333 nmol of forward and reverse primers and KAPA HiFi PCR Master Mix (Kapa Biosystems, Boston, MA, USA). PCR was conducted as follows: an initial 3-min enzyme activation step at 95°C; 20 cycles of 15 s at 98°C, 30 s at 50°C, and 40 s at 72°C; and 10 min at 72°C. Then, the amplification products were cooled to 10°C before purification using clean beads (N411-02, Vazyme Biotech Co., Ltd., Nanjing, China). Index PCRs were performed in a mixture of 15 μl of KAPA HiFi HotStart ReadyMix (Kapa Biosystems, Boston, MA, USA), 1 μl of forward primer, and 1 μl of barcode-primer R X, along with 13 μl of PCR-grade sterile water, to a final volume of 30 μl into the amplification products obtained in the above step. PCR was conducted as follows: an initial 3-min enzyme activation step at 95°C; 5 cycles of denaturation at 95°C for 15 s, annealing at 58°C for 30 s, and extension at 72°C for 40 s; and a final extension step at 95°C for 10 min. Amplification products were analyzed by electrophoresis through a 1% agarose gel containing ethidium bromide and compared to a molecular weight standard (100 bp).

### Sequencing

Following PCR, all amplification products from different samples were mixed in equal concentrations. Sequencing was performed according to the instructions for the Illumina HiSeq-2,500 platform (PE250).

### Bioinformatics Analysis of 16S rRNA Gene Amplicons

#### Raw Data Filtering, Classification, and Annotation

Sequences were trimmed to remove adaptor and PCR primer sequences and binned for a minimal sequence length of 250 bases and an average minimal base quality threshold of 25. The resulting sequences were clustered at 97% identity by QIIME. All of the operational taxonomic units (OTUs) that were lower than four reads in abundance were discarded. Chimera detection and removal were assessed using the GOLD database and the UCHIME algorithm. OTUs were annotated by mothur to the closest taxonomic neighbors according to small subunit ribosomal RNA from SILVA (Quast et al., 2013).

#### Alpha Diversity Analysis

In ecology, alpha diversity is the mean diversity in sites at a local scale. As the richness of the microbiota is affected by the depth of sequencing, the OTU table for each specimen was rarefied using QIIME software (version 1.91) to the same depth, i.e., the number of reads in the specimen with the fewest reads. The Shannon index, which represents richness and evenness of taxa, was estimated using QIIME (version 1.91).

#### Beta Diversity Analysis

Unweighted UniFrac distance matrices were defined based on a profiling table (Lozupone et al., 2011). Weighted UniFrac distance was constructed after determining the unweighted UniFrac distances (Lozupone et al., 2007). PCoA (principal coordinates analysis) was performed based on these data. Intergroup LEfSe (LDA effect size) was performed to reveal biomarkers with significant differences.

### Statistical Analysis

The results are presented as the mean ± SE. The significance of differences was analyzed by one-way ANOVA followed by *post hoc* testing (Bonferroni’s multiple comparison test) using GraphPad Prism version 6.0 for Windows (GraphPad Software, San Diego, CA; www.graphpad.com). Differences were considered statistically significant at *p* < 0.05.

## Results

### Effects of Linggui Zhugan on Body Weight and Body Fat Mass

**Figure 1:**
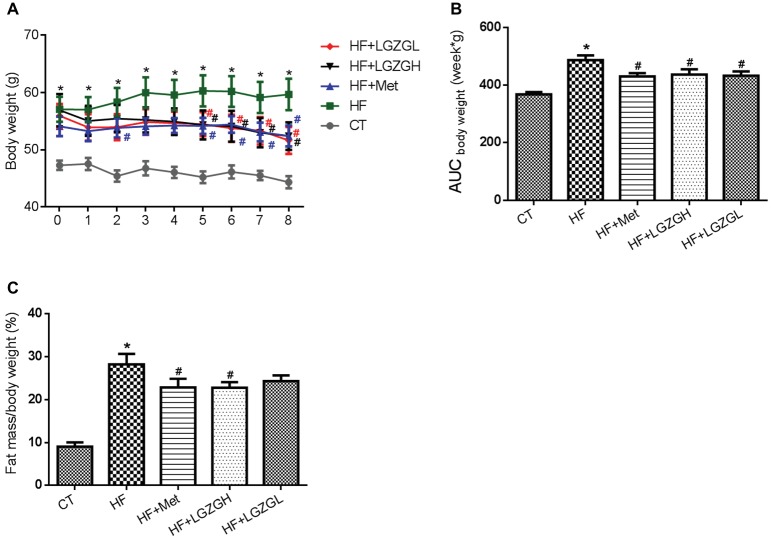
Effect of LGZG on body weight and body fat mass. **(A)** Body weight; **(B)** area under the curve (AUC) of body weight; **(C)** body fat mass. The data are expressed as the mean ± standard error of the mean (*n* = 10). Mice were fed a chow diet (CT), a high-fat diet (HF), a high-fat diet and metformin (HF + Met), a high-fat diet and a high dose of Linggui Zhugan (HF + LGZGH), or a high-fat diet and a low dose of Linggui Zhugan (HF + LGZGL) for 8 weeks. ^*^p < 0.05 compared with the CT group; ^#^*p* < 0.05 compared with the HF group.

Metformin and two dosages of LGZG (3.6 g/kg/d and 1.8 g/kg/d) were administered orally to different groups of HF diet-induced diabetic mice for 8 weeks, and the impact on body weight and body fat mass was determined. At the initial time point, mice fed a HF diet displayed significantly increased body weights compared with those fed a standard chow diet. However, no significant difference in body weight was observed among the mice in the four HF diet groups. Beginning from the fifth week, the body weights of the mice in the HF + LGZGH and HF + LGZGL groups were significantly lower than those of their peers in the HF group (*p* = 0.043, *p* = 0.038, *p* = 0.036, and *p* = 0.013 for the HF + LGZGH group vs. the HF group at weeks five through eight, respectively; *p* = 0.047, *p* = 0.029, *p* = 0.048, and *p* = 0.006 for the HF + LGZGL group vs. the HF group at weeks five through eight, respectively; Figure 1A). The 8-week treatment with either LGZGH or LGZGL markedly reduced body weight gain compared with vehicle treatment (*p* = 0.021 and *p* = 0.013, respectively, Figure 1B). In addition, LGZGH treatment induced a significant decrease in body fat mass in HF diet-fed mice (*p* = 0.0029), while LGZGL treatment tended to reduce body fat mass compared with vehicle treatment plus HF diet, but the difference was not significant (*p* = 0.112, Figure 1C).

### Effects of Linggui Zhugan on Fasting Blood Glucose Levels and HbA1C

**Figure 2:**
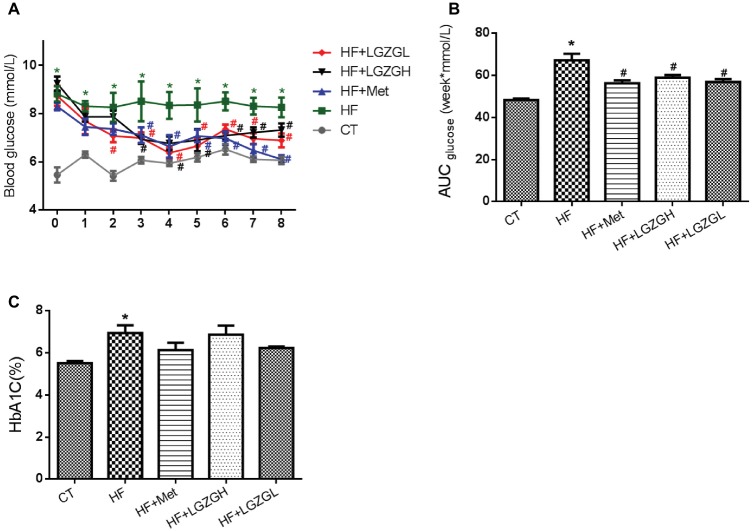
Effect of LGZG on fasting blood glucose levels and HbA1C. **(A)** Fasting blood glucose levels; **(B)** area under the curve (AUC) of fasting blood glucose; **(C)** glycosylated hemoglobin (HbA1C). The data are expressed as the mean ± standard error of the mean (*n* = 10). Mice were fed a chow diet (CT), a high-fat diet (HF), a high-fat diet and metformin (HF + Met), a high-fat diet and a high dose of Linggui Zhugan (HF + LGZGH), or a high-fat diet and a low dose of Linggui Zhugan (HF + LGZGL) for 8 weeks. ^*^p < 0.05 compared with the CT group; ^#^*p* < 0.05 compared with the HF group.

FBG levels were measured weekly. As expected, FBG levels in the HF groups were significantly higher than those in the CT group. Compared with the HF mice, the mice treated with metformin, LGZGH, and LGZGL had lower FBG levels, and a significant difference was observed after the third week (*p* = 0.002 and *p* = 0.000, respectively, Figures 2A,B). Moreover, marked elevations in HbA1C, an important indicator for monitoring long-term blood glucose control, were observed in the HF group compared with the CT group (*p* = 0.001). Notably, metformin, LGZGH, and LGZGL treatment tended to reverse the increase in HbA1C (*p* = 0.070, *p* = 0.871, and *p* = 0.111, respectively; Figure 2C). However, the differences among the HF groups were not significant, possibly because the experimental protocol lasted 8 weeks, and a more prolonged course of drug administration may reveal positive changes in HbA1C measurements.

### Linggui Zhugan Alleviates Glucose Intolerance and Insulin Resistance

**Figure 3:**
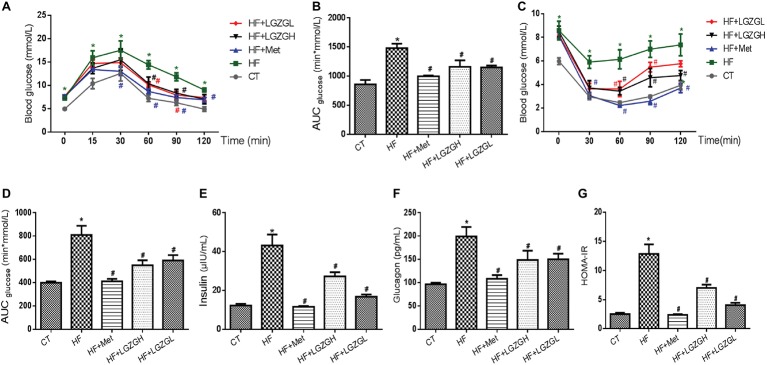
Effect of LGZG on glucose intolerance and insulin resistance. **(A)** Oral glucose tolerance test (OGTT); **(B)** area under the glucose response curve of OGTT; **(C)** insulin tolerance test (ITT); **(D)** area under the glucose response curve of ITT; **(E)** insulin; **(F)** glucagon; **(G)** homeostasis model of insulin resistance (HOMA-IR). The data are expressed as the mean ± standard error of the mean (*n* = 5 in OGTT and ITT; *n* = 10 in insulin, glucagon, and HOMA-IR). Mice were fed a chow diet (CT), a high-fat diet (HF), a high-fat diet and metformin (HF + Met), a high-fat diet and a high dose of Linggui Zhugan (HF + LGZGH), or a high-fat diet and a low dose of Linggui Zhugan (HF + LGZGL) for 8 weeks. ^*^p < 0.05 compared with the CT group; ^#^*p* < 0.05 compared with the HF group.

To validate the effect of LGZG on glycemic homeostasis, the mice were subjected to OGTT and ITT, and the results are presented in Figure 3. We found that a HF diet induced impaired glucose tolerance compared with the control diet based on the OGTT and ITT results. As expected, significantly lower glucose levels were observed in the metformin-, LGZGH-, and LGZGL-treated groups than in the untreated HF group between 60 and 90 min during the OGTT (Figure 3A), along with significantly lower AUC levels (*p* = 0.000, *p* = 0.004, and *p* = 0.004, respectively; Figure 3B). Similarly, significantly lower glucose levels were observed at 30, 60, 90, and 120 min in both the metformin and LGZGH groups, as well as at 30 and 60 min in the LGZGL group, during the ITT (Figure 3C). Notably, treatment with metformin, LGZGH, and LGZGL significantly decreased the AUC in the ITT (*p* = 0.000, *p* = 0.000, and *p* = 0.003, respectively; Figure 3D).

As shown in Figures 3E–G, we also measured plasma insulin and glucagon levels and then calculated HOMA-IR to determine the effect of LGZG on insulin resistance. Plasma insulin and glucagon levels were significantly lower in all treatment groups than in the HF group (*p* = 0.000 for all treatment groups vs. the HF group, Figure 3E; *p* = 0.000, *p* = 0.016, and *p* = 0.019 for the HF + Met group, the HF + LGZGH group, and the HF + LGZGL group, respectively, vs. the HF group, Figure 3F). Furthermore, considerable decreases in HOMA-IR values were observed in the metformin-, LGZGH-, and LGZGL-treated groups relative to the HF group (*p* = 0.000, *p* = 0.000, and *p* = 0.001, respectively; Figure 3G). These results suggested that LGZG could ameliorate impaired glucose tolerance and insulin resistance and delay the onset of hyperglycemia.

### Linggui Zhugan Regulates Serum Lipid Profiles and Free Fatty Acid Levels

**Figure 4:**
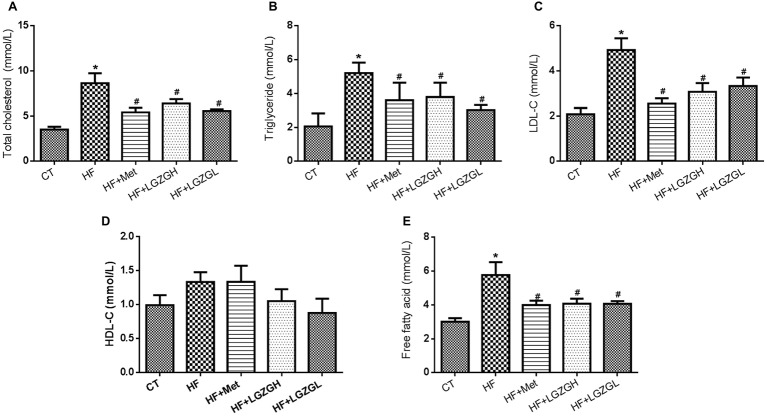
Effect of LGZG on serum lipid profiles and free fatty acid levels. **(A)** Total cholesterol (TC); **(B)** triglyceride (TG); **(C)** low-density lipoprotein cholesterol (HDL-C); **(D)** high-density lipoprotein cholesterol (LDL-C); **(E)** free fatty acid (FFA). The data are expressed as the mean ± standard error of the mean (*n* = 10). Mice were fed a chow diet (CT), a high-fat diet (HF), a high-fat diet and metformin (HF + Met), a high-fat diet and a high dose of Linggui Zhugan (HF + LGZGH), or a high-fat diet and a low dose of Linggui Zhugan (HF + LGZGL) for 8 weeks. ^*^p < 0.05 compared with the CT group; ^#^*p* < 0.05 compared with the HF group.

To further evaluate the ameliorative effects of LGZG on HF diet-fed mice, we analyzed lipid and FFA levels in mouse serum (Figure 4). Plasma TC, TG, and LDL-C levels in the HF groups were significantly elevated compared with those in the CT group. After treatment with LGZG for 8 weeks, serum TC, TG, and LDL-C levels were decreased by 25.84, 27.25, and 37.53%, respectively, in the LGZGH group compared with the HF group (*p* = 0.013, *p* = 0.006, and *p* = 0.001, respectively). In the LGZGL group, serum TC, TG, and LDL-C levels were reduced by 35.51, 41.90, and 32.39%, respectively (*p* = 0.000, *p* = 0.000, and *p* = 0.004, respectively; Figures 4A–C). However, no difference in HDL-C level was observed among the groups (*p* = 0.994 for the HF + Met group vs. the HF group; *p* = 0.291 for the HF + LGZGH group vs. the HF group; *p* = 0.089 for the HF + LGZGL group vs. the HF group; Figure 4D). Moreover, FFA levels were considerably higher in the HF group than in the CT group (*p* = 0.000) and were significantly reduced by metformin and LGZG treatment (*p* = 0.004 for the HF + LGZGH group vs. the HF group; *p* = 0.004 for the HF + LGZGL group vs. the HF group; Figure 4E).

### Analysis of Gut Microbiota in Different Groups Based on 16S rRNAs

**Figure 5:**
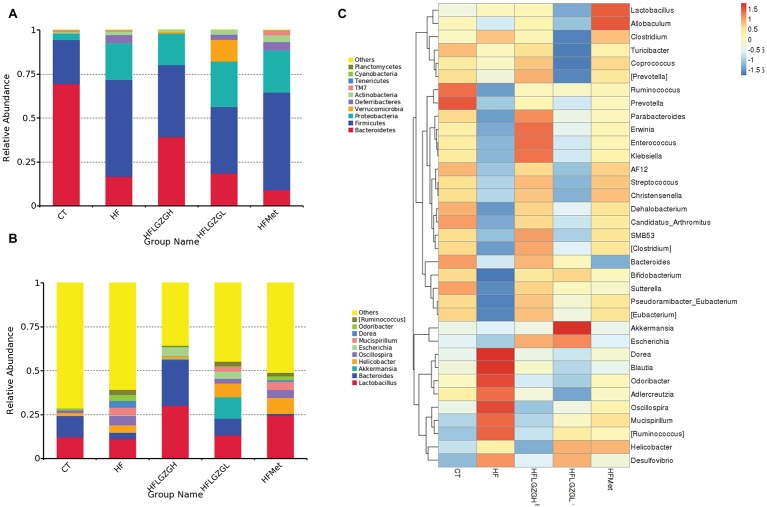
Analysis of phyla abundance in each group. **(A)** Relative abundance distribution of operational taxonomic unit (OTU) sequences at the phylum level from the 10 most prominent bacterial phylotypes; **(B)** relative abundance distribution of OTU sequences at the genus level from the 10 most prominent bacterial phylotypes; **(C)** comparisons of gut metagenomic profiles at the genus level on a heatmap. Mice were fed a chow diet (CT), a high-fat diet (HF), a high-fat diet and metformin (HF + Met), a high-fat diet and a high dose of Linggui Zhugan (HF + LGZGH), or a high-fat diet and a low dose of Linggui Zhugan (HF + LGZGL) for 8 weeks. There were seven mice in the HF + LGZGH group and six mice in each of the other groups.

**Figure 6:**
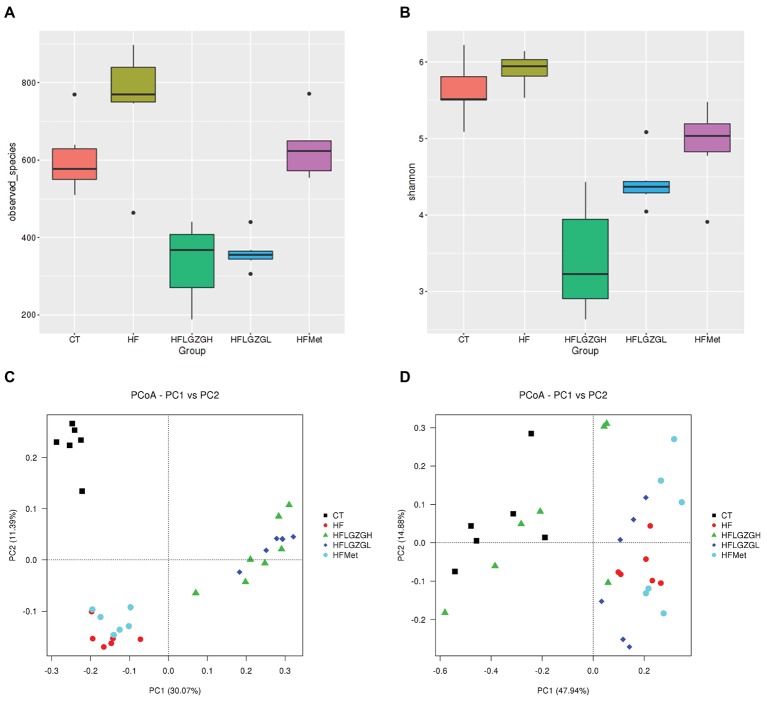
Comparison of richness and diversity (richness and evenness) of the taxa in each group. Alpha diversity analysis, including **(A)** observed taxa and **(B)** Shannon diversity. Principal coordinates analysis (PCoA) of sample clustering results with a weighted UniFrac distance matrix **(C)** and an unweighted UniFrac distance matrix **(D)**. Mice were fed a chow diet (CT), a high-fat diet (HF), a high-fat diet and metformin (HF + Met), a high-fat diet and a high dose of Linggui Zhugan (HF + LGZGH), or a high-fat diet and a low dose of Linggui Zhugan (HF + LGZGL) for 8 weeks. There were seven mice in the HF + LGZGH group and six mice in each of the other groups.

**Figure 7:**
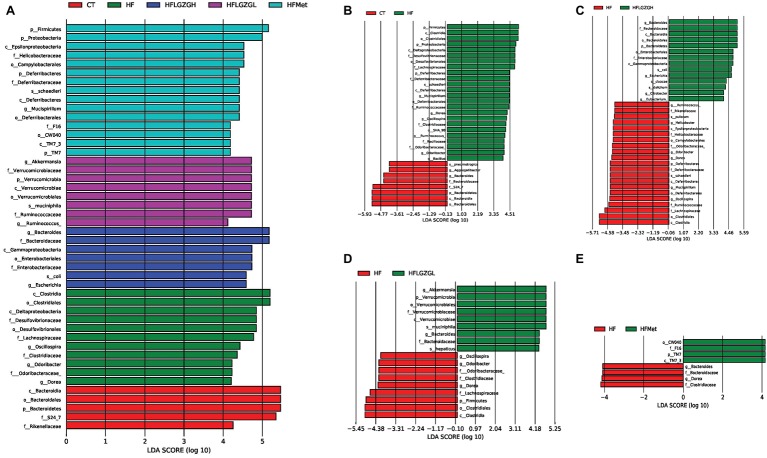
Contribution of gut microbiota to differences among groups. Linear discriminant analysis (LDA) scores of the taxa with significant differences, ranked according to effect size. Discriminant analysis effect size (LEfSe) analysis was applied; taxa with an LDA score > 4 and *p* < 0.05 determined by the Wilcoxon signed-rank test are shown in the Figure. **(A)** Histogram of LDA score comparison among groups. **(B–E)** Histogram of LDA score comparison between the intervention group and the HF diet control group. Mice were fed a chow diet (CT), a high-fat diet (HF), a high-fat diet and metformin (HF + Met), a high-fat diet and a high dose of Linggui Zhugan (HF + LGZGH), or a high-fat diet and a low dose of Linggui Zhugan (HF + LGZGL) for 8 weeks. There were seven mice in the HF + LGZGH group and six mice in each of the other groups.

To assess the impact of LGZG on gut microbiota, we sequenced V3-V4 amplicons of 16S rRNA genes. The raw data can be accessed through the SRA accession PRJNA547497 at NCBI repository. We obtained a total of 4,182,302 (134,913 ± 6,930) high-quality sequences from 31 fecal samples. The effective reads were then clustered into 3,237 OTUs at a similarity cutoff of 97%. After comparing the results with the reference database, we performed bacterial taxonomic identification. Then, based on the results of bacterial taxonomic identification, we conducted the following analyses: alpha diversity (richness and evenness) of a single sample, visualization and significance analysis of beta diversity differences among groups, and screening of taxa with significant differences among groups. The results are shown in Figures 5–7.

#### Analysis of Phyla Abundance in Each Group

The relative phyla abundance (top 10 most prevalent bacteria) in each sample (shown in Supplementary Material S3) and group (Figures 5A,B) was shown at different taxonomic levels.

At the phylum level (Figure 5A), 80–90% of the gut bacterial community present in the feces was dominated by Firmicutes, Bacteroidetes, and Proteobacteria. The relative abundances of Firmicutes in the CT and HF groups were 25.2 and 55.1%, respectively, and the abundances of Bacteroidetes in the CT and HF groups were 69.4 and 16.4%, respectively. After treatment with LGZG, the relative abundances of Firmicutes were significantly decreased (40.8 and 37.7% in the HF + LGZGH and the HF + LGZGL groups, respectively), and those of Bacteroidetes were markedly increased (39.4 and 18.7% in the HF + LGZGH and the HF + LGZGL groups, respectively) compared with those in the HF groups. Accordingly, LGZG treatment restored the HF diet-induced increase in the Firmicutes/Bacteroidetes ratio, a widely used marker of gut dysbiosis.

At the genus level (Figure 5B), although the percentages of *Lactobacillus, Bacteroides, Oscillospira, Helicobacter, Akkermansia, Mucispirillum,* and *Escherichia* were significantly altered after the administration of LGZG or metformin, some bacteria (*Akkermansia, Escherichia,* and *Mucispirillum*) were present in extremely small populations (less than 0.1%). Specifically, both LGZGH and LGZGL significantly increased the abundance of *Lactobacillus* (30.20 and 13.26% in the HF + LGZGH and HF + LGZGL groups, respectively, vs. 11.22% in the HF group) and *Bacteroides* (25.89 and 9.54% in the HF + LGZGH and HF + LGZGL groups, respectively, vs. 3.55% in the HF group) and significantly decreased the abundance of *Oscillospira* (0.12 and 2.74% in the HF + LGZGH and HF + LGZGL groups, respectively, vs. 5.42% in the HF group). In addition, *Helicobacter* was also remarkably decreased in the LGZGH group (1.39 vs. 4.30% in the HF group) but notably increased in the LGZGL group (7.87 vs. 4.30% in the HF group). After metformin treatment, the abundance of *Lactobacillus* (24.46 vs. 11.22% in the HF group) and *Helicobacter* (9.10 vs. 4.30% in the HF group) showed a significant increase.

Furthermore, the taxa with the top 35 relative abundances were clustered in a heat map at the genus level to represent the most prominent ones (Figure 5C). The pheatmap function in the pheatmap package of R language was used, and the function default parameter of *clustering_distance_rows = euclidean distance* in the clustering method was applied in the current study. The results support the above effect of LGZG.

#### Analysis of Gut Microbiota Diversity in Each Group

To assess microbial community composition, the richness and diversity (richness and evenness) of the taxa were calculated. As shown in Figure 6A, the richness (alpha diversity measured by observed taxa estimator) revealed that the gut microbiota composition in the HF group was significantly different from that in the CT group (*p* = 0.028). After 8 weeks, the composition of gut microbiota was significantly changed in the groups treated with LGZGH and LGZGL compared with the HF group (both *p* = 0.000). However, the difference between the metformin and HF groups was not significant (*p* = 0.160). The HF diet induced a slight increase in diversity (alpha diversity represented by the Shannon index) (*p* = 0.118 compared with the control diet), which was reduced by LGZGH, LGZGL and metformin (all *p* = 0.000 compared with vehicle treatment in the HF group, Figure 6B).

The community composition of the microbiota was also analyzed using PCoA, a type of beta diversity analysis, with unweighted and weighted UniFrac distance matrices.

As shown in Figures 6C,D, both weighted and unweighted UniFrac matrices indicated a significant separation among groups, indicating that the HF diet and different treatments were both key factors influencing the composition of gut microbiota. Adonis analysis, a type of nonparametric multivariate analysis of variance, was conducted to evaluate the differences among groups. Significant differences in the first principal component were observed among the CT, HF, HF + Met, HF + LGZGH, and HF + LGZGL groups. Samples from mice fed a HF diet were completely separated from those in the CT group (*p* = 0.005). In addition, the gut flora that was modulated by LGZGH, LGZGL and metformin was separated from that of the HF group (*p* = 0.001 for both the HF + LGZGH and HF + LGZGL groups vs. the HF group, *p* = 0.025 for the HF + Met group vs. the HF group). The clustering tree of the groups using UPGMA (Unweighted Pair-group Method with Arithmetic Mean) was shown in Supplementary Material S4.

#### Analysis of the Microflora Contributes to Differences Among Groups

In addition, the linear discriminant analysis (LDA) and effect size (LEfSe) were used to assess specific changes in gut microbiota among groups. The LEfSe provides a list of taxa depending on the different treatments with statistical and biological significance and ranks them according to effect size (Segata et al., 2011). The taxa with an LDA score higher than 4 and a *p* < 0.05 as determined by the Wilcoxon signed-rank test are shown in Figures 7A–E.

Similar to the results shown in Figure 5, MetaStat analysis showed significant differences in the relative abundance of bacterial taxa in feces among groups at the phylum, class, order, family, and genus levels. At the phylum level, the abundance of Bacteroidetes, Firmicutes, Proteobacteria, Deferribacteres, Verrucomicrobia, and TM7 was significantly different among groups (Figure 7A). At the genus level, there were a total of eight known genera with differential abundance between the HF and CT groups, namely, *Mucispirillum, Dorea, Oscillospira, Odoribacter, Ruminococcus, Bacillus, Bacteroides,* and *Aggregatibacter* (Figure 7B). Figure 7C displays the 10 communities that contributed to the difference in taxa between the HF + LGZGH and HF groups: *Bacteroides, Escherichia, Citrobacter, Eubacterium, Mucispirillum, Ruminococcus, Helicobacter, Odoribacter, Dorea,* and *Oscillospira*. LGZGL selectively affected the following five taxa: *Bacteroides, Oscillospira, Odoribacter, Dorea,* and *Akkermansia* (Figure 7D). However, the percentage of *Akkermansia, Escherichia, Mucispirillum, Dorea, Ruminococcus, Bacillus, Aggregatibacter, Citrobacter,* and *Eubacterium* relative to all bacteria was extremely small (less than 0.1%). Compared with the HF diet, metformin demonstrated a powerful effect on *Bacteroides* and *Dorea* (Figure 7E).

These results indicate that the HF diet and treatment with LGZG and metformin modulated the composition and diversity of the gut microbiota.

## Discussion

Metabolic disorders, such as obesity, metabolic syndrome, T2DM, etc., are attributed to a combination of genetic susceptibility and lifestyle factors and are increasingly prevalent globally. At present, numerous investigations have focused on finding better treatments or ways to prevent and alleviate these diseases. LGZG, a classic Chinese formula with the effects of strengthening the spleen and warming to remove turbidity, is simultaneously administered to prevent and treat metabolic disorders with excessive dampness (Liang and Wang, 2016). Our results demonstrated that LGZG had a positive effect on controlling body weight gain and decreasing the body fat mass of HF diet-induced diabetic mice. Mice fed a HF diet for 12 weeks manifested elevated FBG levels and serum HbA1C and impaired glucose tolerance and insulin sensitivity, which was consistent with previous studies (Raubenheimer et al., 2006; Zuo et al., 2017). In our study, both high and low doses of LGZG reduced FBG levels. This finding may be due to the ability of LGZG to decrease insulin and glucagon levels. According to the HOMA-IR results, the IR index decreased noticeably in the LGZG groups compared with the HF group, suggesting that the potential mechanism underlying glycemic control by LGZG may be associated with improved insulin resistance. The above effects are similar to those of metformin. In addition to regulating glucose metabolism, our results also showed that LGZG could lower serum lipid and FFA levels. Generally, body weight and glycolipid metabolism are interrelated (Koutsari and Jensen, 2006; Gong et al., 2013). LGZG regulates metabolism through its effect on the spleen in Chinese medicine (Huang et al., 2017). Therefore, the ability of LGZG to decrease lipid levels, body fat mass, and body weight is understandable.

There are many indications of a causative role of the gut microbiota in the development of obesity, glucose homeostasis, and insulin resistance (Houghton et al., 2018; Tam et al., 2018). Hence, drugs that modulate the gut microbiota show potential for treating these diseases. Interestingly, most Chinese herbal drugs are prepared in the form of a decoction; therefore, the gut microbiota is a promising area for exploring the underlying mechanisms of Chinese medicine. Notably, LGZG exerts its function through regulating spleen transportation and transformation, which includes most functions of the digestive system in Western medicine. Moreover, according to the functional system in Chinese medicine, the gut microbiota relates closely to the function of the splenic system (Gong et al., 2016). Therefore, we further detected 16S rRNA in feces to explore the effect of LGZG on gut microbiota.

Both the taxonomic and functional composition of the gut microbiota might be linked and contribute to many metabolic disorders, such as obesity and diabetes. Previous studies have indicated that obesity is associated with an increase in the phylum Firmicutes and a relatively lower abundance of the phylum Bacteroidetes (Turnbaugh et al., 2006, 2009). Moreover, the Firmicutes/Bacteroidetes ratio was found to be positively correlated with glucose tolerance and plasma glucose concentration (Brugman et al., 2006; Larsen et al., 2010). The obesity phenotype was successfully replicated through transplantation of intestinal microbiota with a high Firmicutes/Bacteroidetes ratio to germ-free mice. A previous study in diabetic rats confirmed that differences in the Firmicutes/Bacteroidetes ratio partly explain the progression of diabetes (Zhou et al., 2019). All of the above evidence suggests that changes in the abundance of Bacteroidetes and Firmicutes could lead to metabolic disorders (Tilg and Moschen, 2014). Hence, some scholars have proposed that increased Firmicutes/Bacteroidetes and Bacteroidetes/Prevotella ratios may be signature biomarkers for obesity and T2DM (Seo et al., 2015). In the present work, we showed that a HF diet induced a higher Firmicutes/Bacteroidetes ratio, which is in agreement with the above literature. We also observed that the Firmicutes/Bacteroidetes ratio was decreased in groups administered LGZG compared with the HF group. This finding indicated that a reduction in the Firmicutes/Bacteroidetes ratio may contribute to the anti-diabetic effect of LGZG.

We found that LGZG could increase the relative abundance of *Lactobacillus*. An increased abundance of *Lactobacillus* has been reported to increase insulin secretion, and the proportion of *Lactobacillus* negatively correlated with blood glucose levels in T2DM patients (Million et al., 2012; Simon et al., 2015). Therefore, an increase in *Lactobacillus* is believed to be useful for reducing blood glucose levels in T2DM patients. Studies demonstrated that *Lactobacillus* administration was able to achieve glycemic control by enhancing insulin sensitivity and increasing GLUT4 expression and adiponectin production (Kim et al., 2013). Therefore, we propose that *Lactobacillus* may be one of the target bacteria of LGZG to increase insulin sensitivity in diabetic mice.

In addition, our results also showed that LGZG was able to enrich the relative abundance of *Bacteroides*, which is important in SCFA-generating bacteria. Mounting evidence has suggested the involvement of the gut microbiota in the onset and pathogenesis of diabetes through the regulation of energy metabolism and fatty acid synthesis (Gerard and Vidal, 2019). One of the key biological molecular mechanisms underlying the impact of gut microbiota on host glycemic control is SCFA production. SCFAs, including acetate, propionate, and butyrate, are pivotal for rectifying host metabolism and immunity. Studies have shown that SCFAs play a significant role in the treatment of diabetes by influencing the secretion of GLP-1 and PYY, inflammation, etc. (Ejtahed et al., 2016; Morrison and Preston, 2016). Therefore, LGZG may exert its anti-diabetes effect through the modulation of *Bacteroides*. However, how LGZG influences SCFAs still requires further confirmation Moreover, LGZG had a powerful effect on reducing *Oscillospira*. This effect of LGZG could be favorable because it has been previously reported that *Oscillospira* is positively associated with the pathogenesis of diabetes (Krych et al., 2015). *Helicobacter* is thought to be an intestinal pathogenic bacterium. Recent studies have demonstrated that *Helicobacter pylori* infection is closely related to insulin resistance and diabetes (Malamug et al., 2014; Cornejo-Pareja et al., 2019). Interestingly, we found that LGZGH reduced the relative abundance of *Helicobacter*, while LGZGL showed the opposite effect. Glycyrrhizic acid, one of the main active ingredients in LGZG, was reported to suppress *Helicobacter* (Krausse et al., 2004), but the effect of other active ingredients and the interaction of different components in LGZG on *Helicobacter* are unknown. Therefore, the results of our study may highlight the complex interaction of the different components of LGZG.

In addition to changes in specific taxa, the richness and diversity of gut microbiota are often significantly altered by HF diets (Daniel et al., 2014; Liu et al., 2018) or different drug interventions (Li et al., 2017; Song et al., 2017). The gut microbiota is a major environmental factor that can influence metabolic modifications in the host organism (Ley et al., 2006); it is involved in very important aspects of metabolism, such as vitamin metabolism (Ramakrishna, 2013), SCFA metabolism (Kimura et al., 2013), neuropeptide response (Frohlich et al., 2016), and food digestion. Studies have shown that mice without microbiota are resistant to developing obesity and insulin resistance when fed a HF diet (Everard et al., 2014; Kubeck et al., 2016). Therefore, some researchers hold that general alterations of the intestinal gut, rather than a specific microbial taxon, have a direct association with T2DM pathophysiology (Qin et al., 2012). Consistent with the above notion, our results showed that mice with HF diet-induced diabetes displayed general changes in microbial composition. In addition, LGZG influences the microbial composition and the dominant gut microbial taxa. Collectively, these results showed that LGZG modulates the gut microbiota of diabetic mice.

In summary, our findings confirmed that LGZG reduced body weight, decreased serum glucose and lipid profiles, improved glucose tolerance, and modulated the composition and some specific taxa of gut microbiota in HF diet-induced diabetic mice. These results will lay a foundation for exploring the medical value and hypoglycemic mechanism of LGZG decoction.

## Author Contributions

RW and QN designed the experiments. RW, DZ, and RA performed the experiments. ZW and BS analyzed the data. RW, DZ, and QN wrote the paper. RA, ZW, YL, and BS critically revised the manuscript. All authors approved the final version of the manuscript.

### Conflict of Interest Statement

The authors declare that the research was conducted in the absence of any commercial or financial relationships that could be construed as a potential conflict of interest.
