# Supplementary material for: Linggui Zhugan Formula Improves Glucose and Lipid Levels and Alters Gut Microbiota in High-Fat Diet-Induced Diabetic Mice
Source: Front Physiol. 2019 Jul 23;10:918. doi: 10.3389/fphys.2019.00918 (PMC6663968; doi:10.3389/fphys.2019.00918)
Supplement: Supplementary file 1 [file Data_Sheet_1.pdf]

S1

Composition of the standard chow diet and the high-fat diet

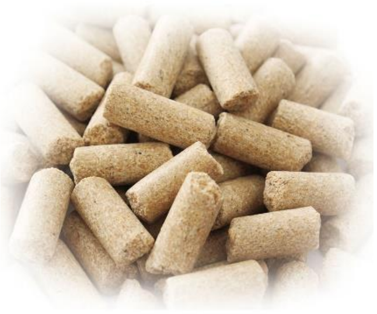

MD12031 (10% Kcal from fat)

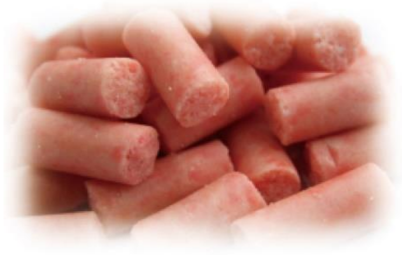

MD12033 (60% Kcal from fat)

Compositions	**MD12031 (10%)**	**MD12033 (60%)**
casein	18.96%	25.84%
L- cystine	0.28%	0.39%
cornstarch	29.86%	0
maltodextrin	3.32%	16.15%
sucrose	33.17%	8.89%
cellulose	4.74%	6.46%
soybean oil	2.37%	3.23%
lard	1.90%	31.66%
compound minerals	4.24%	5.81%
compound vitamins	0.95%	1.29%
choline bitartrate	0.19%	0.26%
total energy (kcal/g)	3.85	5.24
