# Supplementary material for: Linggui Zhugan Formula Improves Glucose and Lipid Levels and Alters Gut Microbiota in High-Fat Diet-Induced Diabetic Mice
Source: Front Physiol. 2019 Jul 23;10:918. doi: 10.3389/fphys.2019.00918 (PMC6663968; doi:10.3389/fphys.2019.00918)
Supplement: Supplementary file 2 [file Data_Sheet_2.pdf]

SHIMADZU
LabSolutions

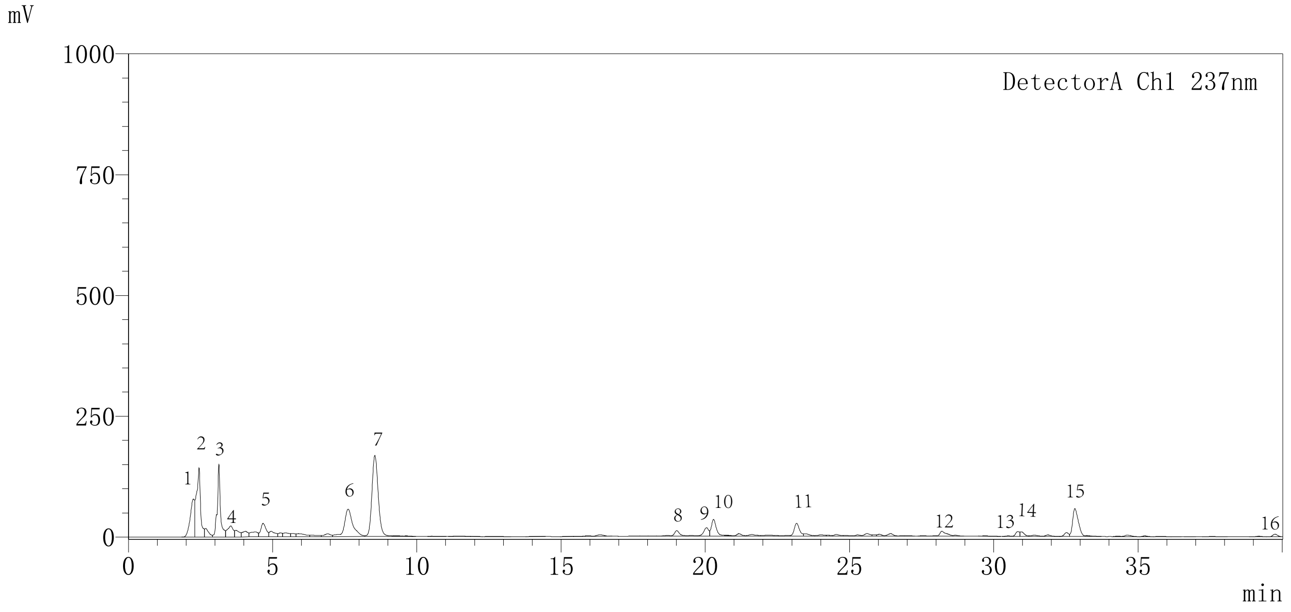

S2 | Fingerprint Chromatogram of Linggui Zhugan. cinnamic acid(6), liquiritin(7), glycyrrhizic acid(15), dehydrotumulosic acid(16).
