# Supplementary material for: Linggui Zhugan Formula Improves Glucose and Lipid Levels and Alters Gut Microbiota in High-Fat Diet-Induced Diabetic Mice
Source: Front Physiol. 2019 Jul 23;10:918. doi: 10.3389/fphys.2019.00918 (PMC6663968; doi:10.3389/fphys.2019.00918)
Supplement: Supplementary file 3 [file Data_Sheet_3.pdf]

S3

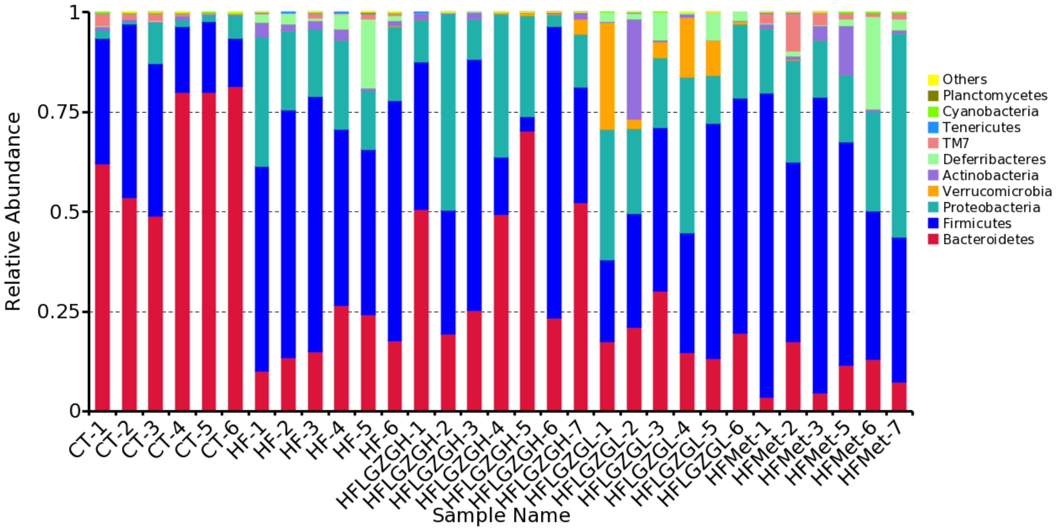

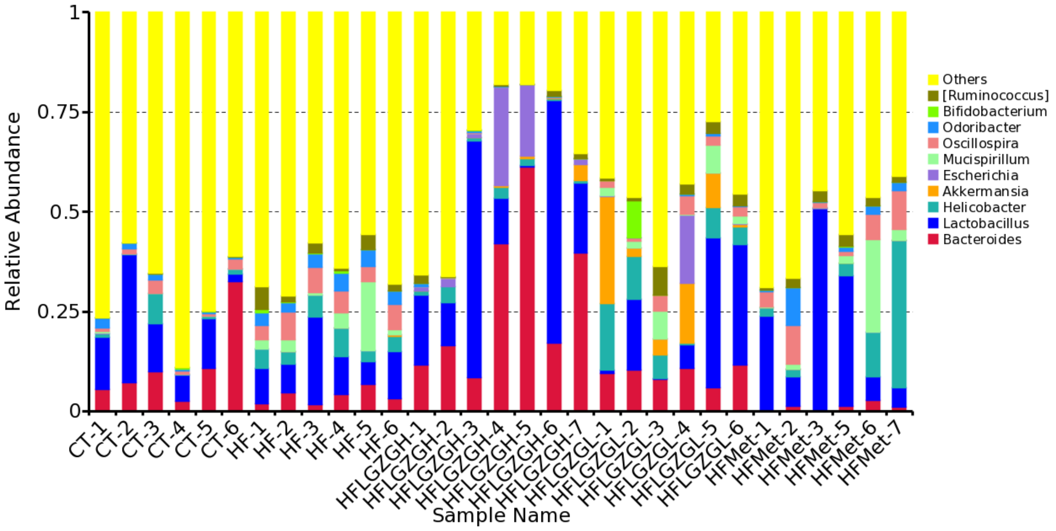

The relative phyla abundance (top 10 detected bacteria) in each sample at the phylum and genus levels
