# Supplementary figures and images for: Linggui Zhugan Formula Improves Glucose and Lipid Levels and Alters Gut Microbiota in High-Fat Diet-Induced Diabetic Mice
Source: Front Physiol. 2019 Jul 23;10:918. doi: 10.3389/fphys.2019.00918 (PMC6663968; doi:10.3389/fphys.2019.00918)

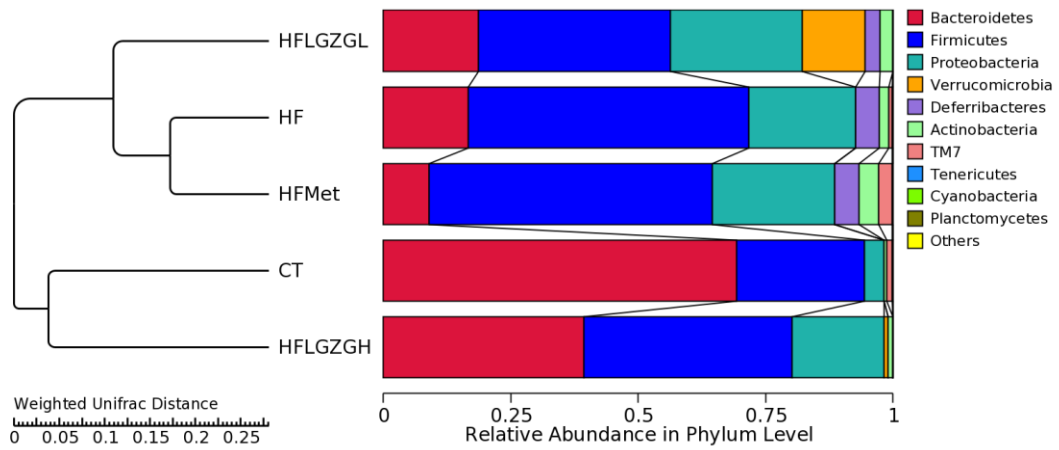

S4 | Clustering tree of the groups using UPGMA (Unweighted Pair-group Method with Arithmetic Mean)

Supplement: Supplementary file 4 [file Data_Sheet_4.pdf]
